# The construction and validation of the Rula wellbeing scale

**DOI:** 10.3389/fpsyg.2026.1876391

**Published:** 2026-10-22

**Authors:** Jeremy J. Coleman, JiSoo Park, Jasmine Davis, Jesse Owen, Chloe Tanega, Allie Woodhouse, Douglas Newton, Sam Seiniger

**Affiliations:** 1 Department of Educational Psychology, Counseling Psychology Program, University of Utah, Salt Lake City, UT, United States; 2 Department of Counseling Psychology, Morgridge College of Education, University of Denver, Denver, CO, United States; 3 Rula Health, Santa Clara, CA, United States

**Keywords:** eudaimonic wellbeing, measurement-based care, positive psychology, psychological wellbeing, psychotherapy, scale development

## Abstract

**Inroduction:**

Psychotherapy outcome research has traditionally emphasized symptom reduction, yet psychotherapy may also enhance eudaimonic wellbeing, including meaning, purpose, connectedness, and adaptive functioning. Despite growing interest in wellbeing as a treatment outcome, few ultra-brief measures are available for routine use in psychotherapy. The present series of studies developed and evaluated the Rula Wellbeing Scale (RWS), a four-item measure designed to assess core domains of eudaimonic wellbeing relevant to clinical practice.

**Methods:**

In Study 1, exploratory factor analysis was used to refine an initial 12-item pool, resulting in a unidimensional four-item solution assessing social connectedness, daily functioning, purpose, and emotion regulation. In Study 2, confirmatory factor analysis in an independent non-clinical sample supported a single-factor structure, whereas a bifactor model was not supported. In both Study 1 & 2, the RWS demonstrated good internal consistency and expected associations with related constructs, including positive correlations with life satisfaction, flourishing, and global wellbeing, and a negative correlation with loneliness. In Study 3, the single-factor structure was replicated in a clinical sample of psychotherapy clients, and the scale demonstrated acceptable internal consistency.

**Results:**

As hypothesized, the RWS scores were negatively associated with depression and anxiety. Mean scores were also lower in the clinical sample than in the non-clinical sample, supporting sensitivity to differences in wellbeing across populations.

**Discussion:**

Collectively, findings provide initial support for the RWS as a psychometrically sound, ultra-brief measure of eudaimonic wellbeing. The scale may have particular utility in psychotherapy and measurement-based care contexts where brief, practical, and conceptually meaningful assessment tools are needed.

## Highlights

- The RWS is a brief 4-item measure of eudaimonic wellbeing designed for use in psychotherapy. This measure is open access and free to use.
- It assesses domains often relevant to treatment progress, including connectedness, purpose, daily functioning, and emotion regulation.
- Findings support its reliability and validity across non-clinical and clinical samples.
- The RWS may be useful in measurement-based care when clinicians want to assess wellbeing alongside symptom change.

## Introduction

Measurement-based care (MBC) is the prevailing method to assessing the effectiveness of psychotherapy process and outcomes (Barkham et al., 2023; Boswell et al., 2023; Bugatti et al., 2023; Brattland et al., 2018; Wampold and Miller, 2023), providing important clinical and research data to understand how clients respond to treatment and benefit from care. Psychotherapy outcome research has traditionally treated symptom reduction as the primary indicator of effective treatment. Yet psychotherapy is also well positioned to promote meaning, purpose, connectedness, and fuller engagement with life (Jankowski et al., 2020; Ryff and Singer, 2003; Seligman, 2011). Indeed, interventions designed to enhance wellbeing show relatively robust effects on wellbeing, whereas evidence for symptom reduction is more variable across intervention types and designs (Bohlmeijer and Westerhof, 2021; Jankowski et al., 2020; Mead et al., 2021; Tulip et al., 2020). A review of positive psychology interventions across 41 countries and 347 studies produced small-to-medium improvements in wellbeing and strengths, alongside meaningful reductions in ratings of depression, anxiety, and stress with interventions effects sustained at three-month follow-up (Carr et al., 2021). Thus, psychotherapy is increasingly conceptualized as having two parallel aims: alleviating distress and fostering wellbeing.

Eudaimonia is a dimension of wellbeing that is especially relevant to psychotherapy (Jankowski et al., 2020; Ruiz et al., 2024). In contrast to hedonic perspectives, which emphasize pleasure, positive affect, and life satisfaction, eudaimonic perspectives emphasize meaning, self-realization, vitality, personal growth, purpose in life, and functioning in accord with valued goals (Ryan and Deci, 2001; Ryff, 1989; Sandage and Hill, 2001). This distinction matters clinically because people may experience some reduction in distress without necessarily regaining a sense of vitality, agency, direction, or coherence in how they are living. In clinical contexts, researchers emphasize that eudaimonic wellbeing can act as both a target of intervention and a valuable index of recovery, often complementing symptom-focused outcomes (Brandel et al., 2017; Bohlmeijer and Westerhof, 2021; Jankowski et al., 2020; Rule et al., 2024). Nevertheless, symptom improvement and eudaimonic improvement should not be presumed to move in lockstep (e.g., Owen et al., 2015). Several reviews note that flourishing tends to integrate hedonic and eudaimonic components, yet scales and research designs vary in how they balance these dimensions; some measures emphasize eudaimonia explicitly, others mix hedonic and eudaimonic elements (Rule et al., 2024; Lin and Chan, 2020; Orth et al., 2022). In short, eudaimonia represents a meaningful domain within a broader, multi-faceted conception of wellbeing relevant to mental health and psychotherapy (Rule et al., 2024; Orth et al., 2022; Lin and Chan, 2020).

Some of the core tenets of MBC have traditionally included outcome monitoring and client feedback regarding the working alliance and responsiveness to treatment. Therapists who are responsive to their MBC feedback show reduced rates of deterioration and superior outcomes compared to those who do not (Delgadillo et al., 2018; Lambert et al., 2018). Over the years, the utilization of ultra-brief measures has increased in an effort balance capturing important treatment data with practical constraints on the data collection process (e.g., time burden, workflow disruptions, inconsistent implementation; Bugatti et al., 2023). Although research and professional guidelines have endorsed MBC as a pathway to higher-quality care, there is a deeper concern that guidelines alone do not automatically translate into practice. This has led to calls for actionable, practice-ready guidelines that address barriers to implementation (e.g., time pressure, workflow disruption) in concrete ways rather than relying on broad recommendations (Bugatti et al., 2023; Wampold and Miller, 2023). This duality, robust evidence alongside uneven real-world adoption, creates a demand for streamlined, practical, and scalable measurement approaches that do not overburden clinicians or patients and still gather important feedback about treatment (Boswell et al., 2023).

Indeed, extensive research on psychological wellbeing has been conducted since its introduction by various scholars (e.g., Deci and Ryan, 1985; Diener, 1984; Ryff, 1989), and numerous measures have been developed to capture what it means to be psychologically well. In a recent review of measures of psychological wellbeing, Koslouski et al. (2022) identified 135 instruments capturing various forms of psychological wellbeing across 49 review articles. The most commonly reported measures in reviews were the Satisfaction with Life Scales (SWLS; Diener et al., 1985) and the World Health Organization Quality of Life Brief Version (WHOQOL-BREF; Skevington et al., 2004). Several scales were also adapted across different cultures and languages, including the Ryff Scales of Psychological Wellbeing (RPWB; Ryff and Keyes, 1995), the SWLS, the McGill Quality of Life Questionnaire (MQOL; Cohen et al., 1995), and the WHOQOL-BREF. While measures introduced in these reviews have generally shown strong reliability and validity, because of their comprehensiveness and length, may not always be useful for repeated use in clinical settings.

Although ultra-brief measures such as the Outcome Rating Scale (ORS; Miller et al., 2003), the Patient Health Questionnaire–4 (PHQ-4; Kroenke et al., 2009), the Working Alliance Inventory–Brief 3 (WAI-B3; Li et al., 2023), and the short-form Cultural Humility Scale (CHS-SF; Coleman et al., 2024) have been developed to assess symptoms, alliance, and humility, few ultra-brief measures provide routine feedback on client wellbeing. One such attempt at an ultra-brief measure of wellbeing was the Rating of Outcome Scale (Seidel et al., 2017). The ROS is a three-item measure of wellbeing which initially demonstrated good reliability, validity, and responsiveness to change. Relative to the ORS, the ROS showed somewhat better concordance with outcomes and less oversensitivity to change, supporting its utility for routine outcome monitoring (Seidel et al., 2017). The ROS captured general wellbeing along three domains (e.g., internal, relational, task-functioning; Seidel et al., 2017), representing a globalized and general psychological wellbeing as opposed to a eudaimonic wellbeing.

### The present study

This study aimed to develop and test the reliability and validity of an ultra-brief wellbeing scale for use in psychotherapy. Informed by theories on eudaimonic wellbeing and MBC literature, we sought to develop a scale with both non-clinical and clinical samples (in psychotherapy) to examine its psychometric evidence for validity and reliability. Further, we plan to establish criterion-related validity via comparison with established psychological wellbeing, depression, and anxiety measures. Finally, we provide guidelines for determining reliable change and clinical cutoff scores. In doing so, the creation, validation, publication, and dissemination of the new Rula Wellbeing Scale (RWS) can address the gap between measuring symptom-reduction versus a holistic view of recovery through creating an ultra-brief open access scale that builds on extensive research in the field of wellbeing while prioritizing the realities of routine clinical care.

## Methods

### Study 1: item selection and exploratory factor analysis with clinical population

#### Participants and procedures

The initial sample (*n* = 475) was selected from a large practice-research network (PRN). From the initial sample, 14 participants were excluded due to invalid responses to any of the validity-check items (e.g., “If you are paying attention, indicate so by choosing response 2”). After screening the data for invalid responses, the analytic sample was n = 461 (see Table 1 for demographics). New clients who had not received any treatment were invited to participate in the study via email communications. Those who elected to participate completed an informed consent form and then completed the measures (see below). All participants were actively seeking mental health care, were 18 years old or older, and were not seeking medication treatment. The treatments were outpatient services provided by licensed therapists across the United States.

**Table 1:** Sample demographics.

Variable	Study 1 (*n* = 461)	Study 2 (*n* = 924)	Study 3 (*n* = 2,757)
Age, M (SD)	36.36 (12.03)	40.85 (12.81)	35.28 (11.07)
Gender			
Woman	74.4%	51.9%	60.7%
Man	24.3%	46.8%	23%
Non-binary	1.3%	1.3%	–
Missing		<0.1%	13.3%
Ethnicity, (%)			
Not Hispanic/Latino/Spanish	79.4%	90.2%	41.9%
Hispanic/Latino/Spanish(other)	20.6%	9.7%	14.3%
Prefer not to Answer	–	0.01%	2.3%
Missing	–	–	41.5%
Race, (%)			
White	51.9%	–*	34.6%
Black or African American	10.4%	–	7.1%
Asian or Asian American	9.6%	–	5.2%
American Indian or Alaskan Native	0.8%	–	0.7%
Native Hawaiian or Pacific Islander	0.4%	–	0.4%
Missing	26.9%		52.7%

This question was not collected due a technical error.

#### Measures

*RULA Wellbeing Scale* (RWS) was tested in this study. See results for psychometric information. The initial item pool consisted of 12 items for the RWS measure designed by the authors to capture multiple theoretically derived domains of wellbeing, across four general domains: (1) social connection/relationships (e.g., I have felt connected to others), (2) daily functioning (e.g., I have been able to keep up with daily responsibilities, like work, school, tasks at home), (3) sense of meaning and purpose (e.g., I have felt a sense of purpose in my life), and (4), regulation (e.g., I have been able to manage my emotions). The scale was developed to assess core domains of eudaimonic wellbeing and adaptive functioning. Items were rated on a 5-point Likert scale anchored as 0 (*strongly disagree*) to 4 (*strongly agree*), with higher scores indicating greater wellbeing and adaptive functioning.

Initially, we sought out therapists within a large practice-research network to understand their opinions and needs in a new wellbeing measure. Based on their feedback and in conjunction with other existing measures, we developed a set of items that were vetted by three expert psychotherapy researchers who have extensive experience with MBC and the theory/practice of eudaimonic wellbeing. They provided open-ended responses to the items. The major theme from the experts was to be mindful of having items that assess various aspects of eudaimonic wellbeing (vs. focusing on a sub-set of items that might be more highly correlated but lack the clinical relevance).

*Schwartz Outcome Scale* (SOS-10; Blais et al., 1999). The SOS-10 is a single dimension, 10-item, brief measure of wellbeing from an intrapersonal and interpersonal perspective (Blais et al., 1999). The SOS-10 utilizes a scale of 0 (*Never*) to 6 (*All/Nearly all of the time*) to evaluate the frequency with which respondents felt in accordance with the respective item (e.g., “I feel hopeful about my future.”, “I am able to handle conflict with others.”). The SOS-10 has shown excellent reliability (*α* = 0.96) across clinical and non-clinical populations. In the present study, the SOS-10 demonstrated excellent reliability, *α* = 0.94.

*The Revised UCLA Loneliness scale – 3 items* (UCLA-3; Hughes et al., 2004; Russell et al., 1980) is a globally used, ultra-brief scale for measuring feelings of social isolation. The three-item scale has demonstrated satisfactory reliability and discriminant validity. The reliability coefficient in the current sample was acceptable, *α* = 0.77.

*Satisfaction with Life Scale* (SLWS; Diener et al., 1985) was used to measure global life satisfaction. The SWLS is a five-item self-report measure of cognitive evaluations of one’s life. Items are rated on a 7-point Likert scale from 1 (*strongly disagree*) to 7 (*strongly agree*), with higher scores suggesting greater life satisfaction. Total scores are calculated by adding item responses. The SWLS has demonstrated strong internal consistency (*α* = 0.87), a unidimensional structure, and convergent validity with related wellbeing constructs (Diener et al., 1985). In the current study, the SWLS demonstrated excellent reliability (*α* = 0.90).

*Moments of Flourishing Experience Scale-16* (MFES-16; Fosha et al., 2025) is a 23-item self-report measure of momentary, positive affect–based flourishing experiences. Items are rated on a 7-point Likert scale 1 (*absolutely untrue of me*) to 7 (*absolutely true of me*), with higher scores indicating greater flourishing. The MFES consists of four subscales: Calm & Self-Acceptance, Vitality, Generosity of Spirit & Gratitude, and Connection & Purpose (Fosha et al., 2025). The psychometric evidence shows a multidimensional structure with strong internal consistency for the total scale (*α* = 0.97) and subscales, as well as convergent and discriminant validity across clinical and general population (Fosha et al., 2025). The current study utilized the 16-item version of the MFES for brevity. The MFES-16 assesses flourishing across three factors (Positive Emotional Wellbeing, Positive Life Functioning, and Positive Interpersonal Relationships). The full-scale MFES-16 demonstrated excellent reliability (*α* = 0.93).

*Generalized Anxiety Disorder-7* (GAD-7; Spitzer et al., 2006). The GAD-7 is a brief clinical assessment of generalized anxiety disorder. Participants are asked to respond to questions about the frequency of anxiety symptoms in the last 2 weeks (Spitzer et al., 2006). The scale uses 4-point Likert scale, from “0” (not at all) to “3” (nearly every day), and the total scores range from 0 to 21. The GAD-7 has established excellent reliability statistics (*α* = 0.92), with good test–retest reliability observed via intraclass correlations (*r* = 0.83; Spitzer et al., 2006). The GAD-7 demonstrated similarly excellent reliability ratings in the current study (*α* = 0.90).

*Patient Health Questionaire-9* (PHQ-9; Kroenke et al., 2001). The PHQ-9 is a brief and widely used measure of depression severity. The PHQ-9 is a self-report measure utilizing a scale of “0” (not at all) to “3” (nearly every day) to assess the frequency with which respondents experience depression symptoms in the past 2 weeks (Kroenke et al., 2001). The total scores range from 0 to 27. In its initial validation, the internal reliability of the PHQ-9 has been established as excellent across two clinical samples (*α* = 0.89, and *α* = 0.86). In the current study, the PHQ-9 indicated excellent (*α* = 0.87).

#### Data analysis

Exploratory factor analysis (EFA) procedures were conducted using IBM SPSS Version 29.0 (IBM Corporation, 2023) to examine the latent structure of the initial item pool and to inform item selection for scale refinement. Because the goal of this phase of scale development was to identify latent constructs rather than simply reduce data, principal axis factoring was used as the extraction method. Given that domains of wellbeing were expected to be obliquely related rather than orthogonal, a direct-oblimin rotation was used.

Factor retention decisions were based on multiple criteria, including eigenvalues (>1), inspection of the scree plot, interpretability of the factor solution, and magnitude and pattern of factor loadings. Items were evaluated for retention based on conceptual relevance, primary factor loadings, and cross-loadings. In line with common recommendations for exploratory factor analysis in scale development, items with primary loadings of approximately 0.30 or higher and minimal cross-loadings (+/− 0.15; Worthington and Whittaker, 2006) were considered for retention, although conceptual considerations and content coverage were also prioritized.

## Study 1: results and discussion

The dataset was examined for missing data. All variables were fully observed; therefore, no missing data analysis (e.g., Little’s MCAR test) was required. Examination of univariate outliers for scores showed that there were no cases in which skewness and kurtosis values were < |2| (George and Mallery, 2010). Additionally, survey responses were subjected to a Kaiser-Meyer-Olkin (KMO) test of sampling adequacy (KMO = 0.922 indicating highest range of accuracy) and Bartlett’s test of sphericity (*p* = <0.001) to determine the appropriateness of further factor analyses, which suggests the data were appropriate to analyze.

Direct-oblimin rotation was used for the initial EFA, given it was expected that factors extracted would be correlated. Additionally, item retention was based on several criteria, including factor loadings ≥ 0.3, absolute factor loadings no greater than 0.3 on more than one factor, item cross-loadings no greater than |0.15|, item communalities ≥ 0.40, and interpretability of factors (Worthington and Whittaker, 2006). Eigenvalue analysis (eigenvalues ≥ 1) indicated the presence of 1 distinct factor. Items with low communalities and weak factor loadings were excluded. The final result was a unidimensional scale consisting of four items and a single-factor solution accounting for 59% of variance, with higher scores indicating stronger experiences of wellbeing across the following dimensions: connectedness, daily functioning, purpose, and emotion regulation (see Table 2 for factor loading estimates).

**Table 2:** Study 1 exploratory factor analysis (clinical sample).

Items	Factor loadings	M (SD)
I have felt a sense of purpose in my life	0.817	2.23 (1.08)
I have felt a sense of direction in my life	0.769	2.05 (1.12)
I have felt that my life has meaning or significance	0.768	2.44 (1.07)
I have been able to have fun	0.725	2.38 (0.99)
I have felt connected to others	0.711	2.40 (1.04)
My relationships have been as satisfying as I want them to be	0.708	1.82 (1.28)
I have been able to appreciate positive things in my life, even small ones	0.681	2.70 (0.86)
I have felt confident in my ability to handle life’s challenges	0.679	2.08 (1.07)
I have felt that I matter to others	0.671	2.71 (0.98)
I have experienced moments of happiness with others	0.648	3.00 (0.79)
I have been able to manage my emotions	0.610	1.84 (1.10)
I have been able to keep up with daily responsibilities & routines (like work, school, or tasks at home)	0.586	2.15 (1.90)

*n* = 461, M, mean; SD, Standard Deviation.

To refine the initial 12-item pool resulted in the development of the RULA Wellbeing Scale (RWS) scale, which demonstrated promising construct validity and reliability. Given these results, we concluded that all items were accessing the same construct. We then calculated Pearson correlations between each retained item and each of the eight excluded items to examine the extent to which the four retained items represented content from the original item pool. All 32 correlations were positive and statistically significant (*p* < 0.001). The retained connectedness item was most strongly associated with experiencing happiness with others (*r* = 0.592), mattering to others (*r* = 0.575), and relationship satisfaction (*r* = 0.542). The daily-functioning item was most strongly associated with having fun (*r* = 0.506) and confidence in handling life’s challenges (*r* = 0.485). The retained purpose item was strongly associated with having a sense of direction (*r* = 0.813) and experiencing life as meaningful or significant (*r* = 0.791). Finally, the emotion-regulation item was most strongly associated with confidence in handling life’s challenges (*r* = 0.572). These correlations indicate that the retained items correspond meaningfully with conceptually related content from the original item pool while preserving coverage of distinct aspects of wellbeing.

Next, we examined how well the four retained items represented the original item pool by examining the relationship between the four-item RWS total and the total score of the eight excluded items. The two scores were strongly positively associated (*r* = 0.844; *p* < 0.001), indicating substantial correspondence (approximately 71% of shared variance) between the retained items and the broader content represented in the original pool. The four-item RWS total was also strongly correlated with the original 12-item total (*r* = 0.935; *p* < 0.001). However, since the four retained items are included in the 12-item total, this association was interpreted descriptively as evidence of a part-to-whole score correspondence rather than as an independent validity estimate.

Additionally, we examined the larger pool of items to reduce to 4 items, with a focus on overall clinical representativeness as well as following the expert advice to keep the items representative of the larger construct. Although the daily-functioning and emotion-regulation items had comparatively lower loadings, their loadings were adequate for retention, and they represented clinically important content not captured by stronger-loading items. Simply, we wanted at least one item per domain that reflects important milestones in clinical work. These four items included: “I have felt connected to others.”, “I have been able to keep up with daily responsibilities & routines (like work, school, or tasks at home).”, “I have felt a sense of purpose in my life.”, and “I have been able to manage my emotions.” The scale reliability was good (*α* = 0.79 especially for a four-item measure).

Based on these four items, consistent with our expectations, the RWS demonstrated a coherent and theoretically expected pattern of associations with the external validity measures (see Table 3). Specifically, RWS scores were positively and strongly associated with other indicators of wellbeing and adaptive functioning, including the SOS-10 (*r* = 0.724), SWLS (*r* = 0.636), and the MFES-16 (*r* = 0.788), suggesting strong convergent validity. In contrast, RWS scores were negatively associated with loneliness (*r* = −0.466), anxiety (*r* = −0.704), and depression (*r* = −0.617), indicating that higher wellbeing as assessed by the RWS corresponds with lower psychological distress and social disconnection. Notably, the strongest associations emerged with conceptually proximal positive flourishing measure (MFES-16), whereas the association with loneliness was more moderate, which is consistent with the view that the RWS captures a broader eudaimonic wellbeing construct rather than merely the absence of psychological distress. Overall, this pattern supports the validity of the RWS as a brief indicator of eudaimonic wellbeing with meaningful, but not redundant, overlap with related constructs. For our next study, we tested the factor structure in a non-clinical sample as well as testing the associations between the RWS and other similar/different constructs.

**Table 3:** Full-scale correlations with RWS.

Measure	Study 1	Study 2	Study 3
SWLS	0.636	0.567**	–
SOS-10	0.724	0.846**	–
UCLA-3	−0.466	−0.506**	–
MFES-16	0.788	0.695	–
PHQ-9	−0.617	–	−0.634
GAD-7	−0.704	–	−0.518

**Correlation is significant at the <0.01 level. Study 1 *n* = 461; Study 2 *n* = 924; Study 3 *n* = 2,757; SWLS, Satisfaction with Life Scale; SOS-10, Schwartz Outcome Scale; UCLA-3, UCLA 3-item Loneliness Scale; MFES-16, 16-item Moments of Flourishing Experience Scale; PHQ-9, Patient Health Questionnaire; GAD-7, Generalized Anxiety Disorder.

## Study 2: confirmatory factor analysis, reliability, validity with non-clinical population

### Participants and procedures

Participants were recruited using CloudResearch (Chandler et al., 2019) survey participant recruitment platform. Research has shown that recruitment from cloud-sourced samples is often more diverse and outperforms pool participants on online attention checks, suggesting a higher level of attentiveness (Chandler et al., 2019; Hauser et al., 2022). Nevertheless, the use of cloud-sourced samples in social science research presents several concerns and limitations. In particular, the potential for invalid data and misrepresentation necessitates the inclusion of screening questions designed to assess participant attentiveness (Burnette et al., 2021). In the present study, three validity checks were employed, and participation was restricted to individuals residing in the United States. The survey took approximately 10–15 min to complete. Upon completion, participants were paid $2.50 amount. From the initial sample (N = 950), 26 participants were excluded due to invalid responses to any of the validity-check items. The final sample consisted of 924 participants (see Table 1 for demographics).

### Measures

*RULA Wellbeing Scale* (RWS) was established in Study 1 as a reliable measure of wellbeing in a single-factor solution. All four items were included in the present study. In the current study, the RWS demonstrated a good reliability coefficient of *α* = 0.83.

*Schwartz Outcome Scale* (SOS-10; Blais et al., 1999). The psychometric properties of the SOS-10 are described in Study 1. In the present study, the SOS-10 demonstrated an excellent reliability coefficient of *α* = 0.95.

*The Revised UCLA Loneliness scale – 3 items* (UCLA-3; Hughes et al., 2004; Russell et al., 1980). The psychometric properties of the UCLA-3 are described in Study 1. The reliability coefficient in the current sample was good *α* = 0.83.

*Satisfaction with Life Scale* (SLWS; Diener et al., 1985). The psychometric properties of the SWLS are described in Study 1. In the current study, the SWLS demonstrated excellent reliability (*α* = 0.94).

*Moments of Flourishing Experience Scale-16* (MFES-16; Fosha et al., 2025) The psychometric properties of the MFES-16 are described in Study 1. In the current study, the MFES-16 demonstrated excellent reliability (*α* = 0.94).

### Data analysis

The purpose of Study 2 was to test the model-fit of the 4-item, single-factor structure of the newly formed RWS with a non-clinical sample and to test the construct validity of the RWS with other measures of mental health. The fit of two different models was tested and compared, including a single-factor model and bifactor model. In the single-factor model items were specified to load onto the associated factor, and each of the four RWS items assessed four aspects of wellbeing. This model suggests that each of the RWS items are related to each other, but each constitutes a distinct feature of wellbeing loading onto a single factor. Conceptually, the bifactor model suggests that a general RWS factor shares variance with all four of the items, and that items with similar content share variance beyond the general factor (Rodriguez et al., 2016).

More specifically, study 2 tested the model fit of the single-factor structure, and a bifactor structure. CFA analyses were conducted using structural equation modeling (SEM) via AMOS version 29 (Arbuckle, 2022). Model fit was evaluated using multiple indices, including the comparative fit index (CFI), root mean square error of approximation (RMSEA), and standardized root mean residual (SRMR) to determine the adequacy of model-to-data fit. Recommended values for these fit indices have ranged from less (CFI ≥ 0.90; RMSEA ≤ 0.08; SRMR ≤ 0.10) to more conservative (CFI ≥ 0.95; RMSEA ≤ 0.06; SRMR ≤ 0.06; Kline, 2016). Notably, and given the low degrees of freedom associated with the model, RMSEA values were interpreted with caution, as RMSEA is known to be inflated and less reliable in models with very few degrees of freedom (Kenny et al., 2015).

### Results and discussion

Study 2 adhered to the same data screening and cleaning procedures outlined in previous studies (see above), with no noteworthy outliers, violations of normality, and with missingness qualified as MCAR. We utilized the same testing conventions as described in Study 1.

Two candidate models were tested (e.g., single-factor and bifactor). The single-factor was tested and observed to fit the data as follows: *x*^2^ = 67.271, *p* < 0.001, *df* = 2; CFI = 0.955; RMSEA = 0.185 (90% CI = 0.149, 0.225), and SRMR = 0.0391. These fit statistics demonstrate good fit to the data, with the exception of RMSEA. RMSEA can be less reliable and inflated in models with very few degrees of freedom (i.e., ultra-brief scales) and should be interpreted alongside the other fit statistics (Kenny et al., 2015). The bifactor model was also evaluated to assess dimensionality and test whether the RWS items simultaneously reflected a general wellbeing factor and more specific domains. However, the model was not identified (*df* = −2), and several parameters, including factor loadings and variances, were not estimable. These results indicate that the bifactor specification was not supported by the data, likely due to the small number of items relative to the model’s complexity. Given the absence of a stable solution and the lack of evidence for meaningful multidimensional structure, the bifactor model was not retained. Instead, the single-factor model was selected as the most parsimonious and empirically supported representation of the RWS.

Our results supported a single-factor model with four items. The highest loading on the general factor was 0.788 for Item 3 (“I have felt a sense of purpose in my life”) followed by Item 1 (“I have felt connected to others”; 0.733), Item 2 ([“I have been able to keep up with daily responsibilities & routines…]”; 0.733), and Item 4 (“I have been able to manage my emotions”; 0.729; see Table 4). Items associated with connectedness, daily functioning, purpose, and emotion regulation were included in this overall factor (see Figure 1). The RWS full-scale reliability was also supported in the CFA results (*α* = 0.832).

**Table 4:** Standardized factor loadings: confirmatory factor analyses.

Item number	Item content	Study 2 *β*	Study 3 *β*
1	I have felt connected to others	0.733	0.621
2	I have been able to keep up with daily responsibilities & routines (work, school, or tasks at home)	0.733	0.534
3	I have felt a sense of purpose in my life	0.788	0.666
4	I have been able to manage my emotions	0.729	0.601

Study 1 *n* = 461; Study 2 *n* = 924; Study 3 *n* = 2,757; *β*, Standardized Factor Loading.

**Figure 1:**
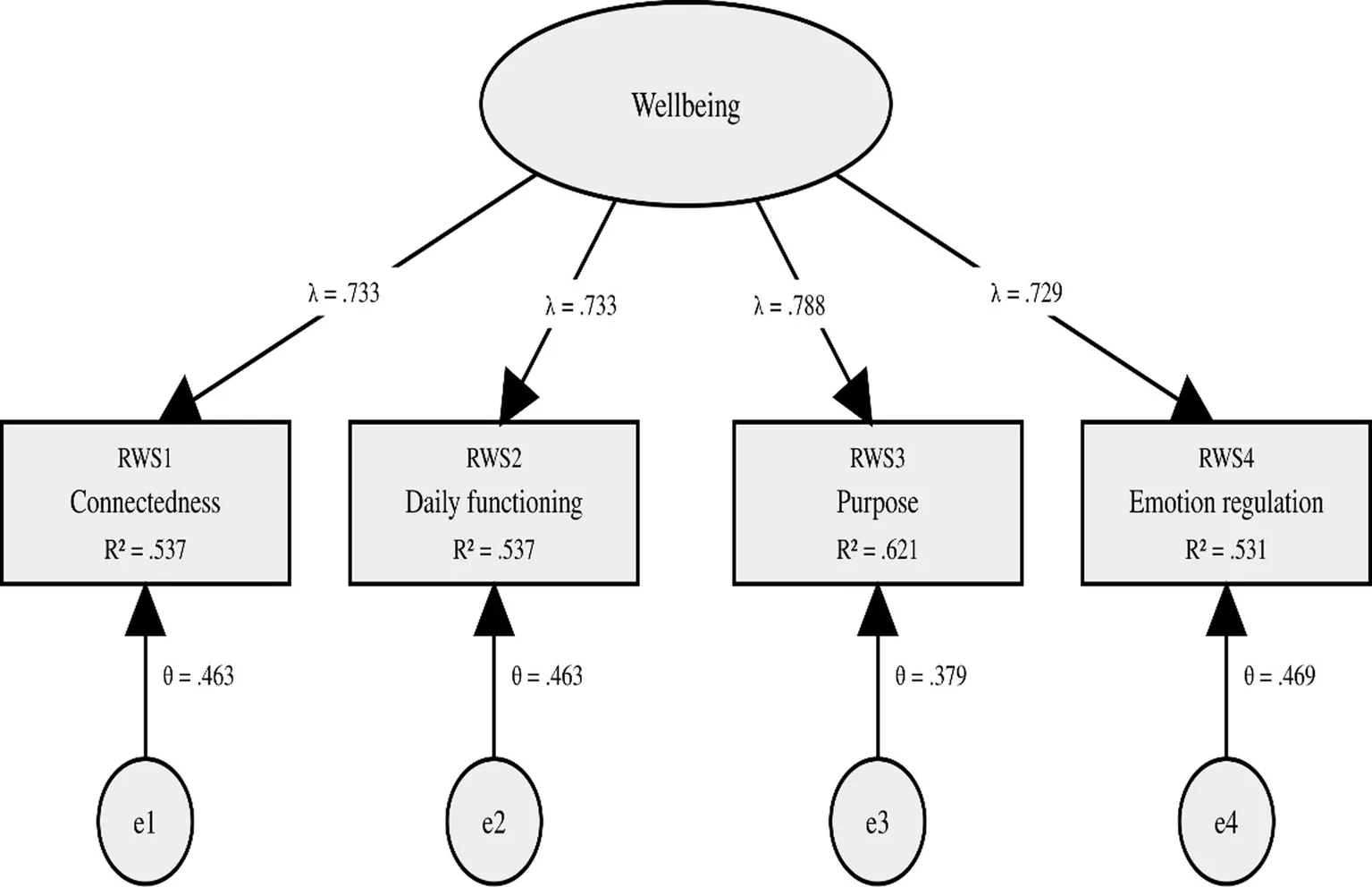
Standardized single-factor model of RWS in Study 2 (non-clinical). λ = Standardized factor loadings expressed as Lambda; *R*^2^ = Squared multiple correlations expressed as *R*-squared, and representing the proportion of variance explained by the latent factor; *θ* = Standardized residuals expressed as Theta. *N* = 924; *x*^2^ = 67.271, *p* < 0.001, *df* = 2; CFI = 0.955; RMSEA = 0.185 (90% CI = 0.149, 0.225), and SRMR = 0.0391.

Finally, as we predicted, the RWS was significantly and negatively correlated with the UCLA three-item loneliness scale (*r* = −0.51, *p* < 0.001). Conversely, the RWS was observed to have significant and positively correlated relationships with the SWLS (*r* = 0.57, *p* < 0.001), SOS-10 (*r* = 0.85, *p* < 0.001), and the MFES-16 (*r* = 0.70, *p* < 0.001). The RWS was moderately and negatively related with loneliness ratings, and a reduced sense of connectedness with others, thus establishing some inverse associations with loneliness alongside convergent validity with measures of wellbeing and flourishing (see Figure 2).

**Figure 2:**
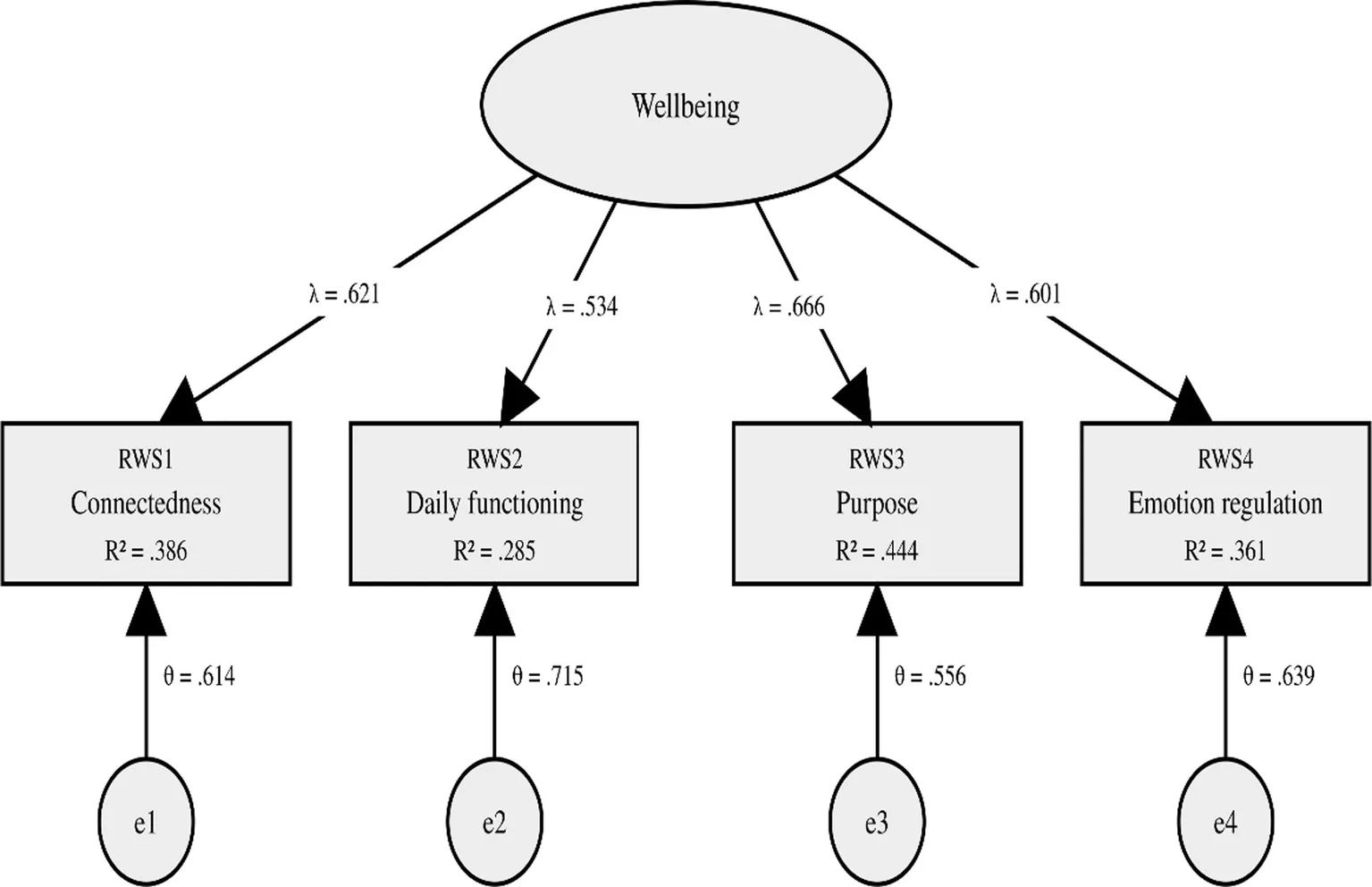
Study 3 standardized single-factor model of RWS in Study 3 (clinical). λ = Standardized factor loadings expressed as Lambda; *R*^2^ = Squared multiple correlations expressed as *R*-squared, and representing the proportion of variance explained by the latent factor; *θ* = Standardized residuals expressed as Theta. *N* = 2,757; *x*^2^ = 19.730, *p* < 0.001, *df* = 2; CFI = 0.990; RMSEA = 0.057 (90% CI = 0.036, 0.081), and SRMR = 0.017.

## Study 3: testing RWS in a clinical sample

The purpose of study 3 was to examine the validity and reliability of the RWS in a clinical sample. The current study includes responses from clients in outpatient treatment settings. The outpatient sample comprised individuals seeking psychotherapy via telehealth services in North America via a PRN. These clients were distinct from the sample in study 1. All clients in the clinical sample entered the treatment program, and research voluntarily. The current study seeks to offer more evidence of construct validity by including measures of pathology (anxiety and depression). We predict that the RULA will be inversely correlated with these measures.

### Participants and procedure

A total of 2,757 participants in Study 3 were recruited from outpatient telehealth psychotherapy. See Table 1 for demographic information.

### Measures

*RULA Wellbeing Scale* (RWS). The psychometric properties of the RWS were established in non-clinical populations in studies 1 and 2. In the current clinical sample, the reliability coefficient was observed to be acceptable (*α* = 0.70).

*Patient Health Questionnaire* (PHQ-9; Kroenke et al., 2001). The psychometric properties of the PHQ-9 are described in Study 1. In the current study, the PHQ-9 indicated good reliability (*α* = 0.87).

*Generalized Anxiety* (GAD-7; Spitzer et al., 2006). The psychometric properties of the GAD-7 are described in Study 1. The GAD-7 demonstrated similar reliability ratings in the current study (*α* = 0.89).

### Results and discussion

The clinical sample had no noteworthy outliers, violations of normality, and no missingness to report. Study 3 offered further support for the reliability of the RWS in a clinical population, with acceptable internal consistency ratings for the full scale (*α* = 0.70). The highest standardized factor loading on the general factor was 0.666 for Item 3 followed by Item 1 (0.621), Item 4 (0.601), and Item 2 (0.534; see Table 4). The single-factor was tested again and observed to fit to the data as follows *x*^2^ = 19.730, *p* < 0.001, *df* = 2; CFI = 0.990; RMSEA = 0.057 (90% CI = 0.036, 0.081), and SRMR = 0.017. These fit statistics demonstrate good fit to the data, suggesting the one factor model is appropriate. Similar to our prediction, the RWS was negatively correlated with the PHQ-9 (*r* = −0.634, *p* < 0.001) and GAD-7 (*r* = −0.518, *p* < 0.001). In other words, the RWS was moderately and negatively associated with both depression and anxiety ratings, which supports expected inverse associations with other measures of psychological disorders.

## Establishing clinical cut-off and reliable change scores

Because the intended use of the RWS includes psychotherapy and outcome-monitoring applications, it was important to establish benchmarks for clinical interpretation. Thus, we evaluated both a clinical cut-off, to distinguish individuals more similar to clinical versus non-clinical populations. The clinical cut-off point is the point in the distribution of both samples with the greatest divergence (minimal overlap).^1^ In doing so, therapists can understand relative to this norming sample if their clients’ functioning has shifted in a more functional range. We also calculated a reliable change index (RCI) score to assist therapists understand the minimum amount of change needed to conclude that score differences are unlikely to be attributable to measurement error alone (cf. Jacobson and Truax, 1991). Establishing these values strengthens the scale’s applied relevance by supporting interpretation of both clinical status and meaningful change over time.

We utilized the non-clinical samples and only selected those who reported less distress on the PHQ-9 & GAD-7 (i.e., under 10) to ensure the non-clinical sample were not significantly distressed. We compared those participants with the combined clinical sample’s RWS scores. The RWS scores range from 0 to 16 and based on the clinical cut-off score the reliable difference between the clinical sample and the non-distress sample was estimated to be 12. This suggests those who score under 12 are more likely to resemble clinically seeking individuals. We also calculated the RCI, or how much change on the scale is needed to rule out measurement error. We utilized a pooled standard deviation, and reliability estimates across the samples to calculate the RCI. The RCI was estimated to be 2.27 (or for simplicity 2 or 2.5), indicating that change of approximately 2–2.5 points can be interpreted as reliable change.

## Overall results

The primary purpose of the present investigation was to develop and establish the psychometric properties of a new ultra-brief measure of eudaimonic wellbeing. Using robust non-clinical and clinical samples in North America, the 4-item RWS was developed. EFA procedures reduced the original 12-item pool to a 4-item single-factor solution with good internal reliability. Subsequent CFA procedures tested two candidate models, a single and bifactor solution, with data fitting the single-factor model more parsimoniously. The ultra-brief RWS efficiently evaluates eudaimonic wellbeing across four domains: connectedness, daily functioning, purpose, and emotion regulation. Overall, the factor structure and reliability of the RWS, yielded robust psychometric properties across non-clinical and clinical samples.

Noteworthy differences in RULA mean scores were found between non-clinical participants in Study 2 and clinical samples in Study 3. Specifically, means for RWS total scores from clinical sample (*M* = 8.25, *SD* = 3.19) were generally lower than non-clinical sample (*M* = 12.89, *SD* = 3.81). Additionally, criterion-based validity was assessed across conceptually related measures. Convergent validity was established by comparing RWS with established positive psychology measures and measures of psychological distress.

## Discussion

The present series of studies aimed to develop and evaluate the psychometric properties of the RULA Wellbeing Scale (RWS), an ultra-brief measure of eudaimonic wellbeing. The four items reflect a unified construct of wellbeing that integrates key domains of functioning, purpose, connection, and emotional regulation. The aim of this scale is to provide a free, open access, psychometrically sound clinical tool with low burden for clients and providers to support treatment planning and assess progress across treatment, promoting collaborative care.

Across three studies, we implemented a systematic, multi-phase scale development process to reduce the item pool, detect and establish the underlying latent structure, and evaluate the reliability and validity of the measure. This included the use of complementary exploratory and confirmatory factor analytic approaches, alongside tests of construct validity in both non-clinical and clinical samples. Collectively, these studies provide a comprehensive and methodologically robust foundation supporting the RWS as an ultra-brief, psychometrically-sound, and empirically grounded indicator of eudaimonic wellbeing.

In Study 1, exploratory factor analytic procedures were used to examine the underlying structure of an initial item pool designed to capture multiple theoretically-derived domains of wellbeing. Using principal axis factoring with oblique rotation, results supported a unidimensional structure, leading to the refinement of the scale from 12 items to a concise 4-item solution. This final structure demonstrated good internal consistency (*α* = 0.79) and conceptual coherence, with items reflecting key domains of wellbeing, including connectedness, daily functioning, purpose, and emotion regulation.

Study 2 sought to confirm this structure and further evaluate the construct validity of the RWS in an independent non-clinical sample. Confirmatory factor analyses supported the single-factor model as the most parsimonious representation of the data, whereas a bifactor model was not empirically supported. Evidence of construct validity was demonstrated through expected associations with established measures of wellbeing and loneliness, indicating that the RWS reliably captures (*α* = 0.83) a general dimension of eudaimonic wellbeing that is related to, but distinct from, both positive functioning and social isolation.

Study 3 extended these findings to a clinical population of individuals engaged in outpatient psychotherapy. The single-factor structure was again supported, and the scale demonstrated acceptable reliability (*α* = 0.70). Consistent with theoretical expectations, RWS scores were negatively associated with measures of depression and anxiety, providing additional evidence of hypothesis-consistent associations with psychological distress in a clinical context. Notably, mean differences between clinical and non-clinical samples further supported the scale’s sensitivity to variations in eudaimonic wellbeing across populations. Finally, we established preliminary clinical interpretive benchmarks for the RWS, extending its utility beyond psychometric evaluation alone. Specifically, the clinical cut-off and RCI estimates suggest the scale may be useful not only for identifying individuals whose scores more closely resemble treatment-seeking populations, but also for detecting meaningful changes in wellbeing over time that are unlikely to reflect measurement error.

Our findings offer initial support for the RWS as a parsimonious and psychometrically-sound indicator of eudaimonic wellbeing. Notably, the emergence and replication of a single-factor structure across studies suggests that the construct assessed by the RWS may reflect a higher-order, integrative dimension of wellbeing that cuts across traditionally distinct domains assessed by the four items (e.g., connectedness, daily functioning, purpose, and emotion regulation). This is consistent with theoretical perspectives that conceptualize mental health not as the absence of pathology, but as the presence of adaptive functioning and positive psychological processes that cohere into a unified experience of wellbeing (Jankowski et al., 2020; VanderWeele et al., 2019). At the same time, the significant and moderate associations observed with both measures of positive functioning (e.g., life satisfaction, flourishing) and indicators of distress (e.g., depression, anxiety, loneliness) suggest that the RWS occupies a meaningful position within this broader nomological network. In other words, the RWS captures a construct that is related to, but not reducible to, either domain alone. Perhaps most importantly, the brevity of the scale, coupled with its consistent performance across non-clinical and clinical samples, highlights its potential utility as a pragmatic tool for both research and applied settings, particularly in contexts where assessment burden may be minimized without sacrificing conceptual coverage.

This measure can provide an opportunity for routine outcome monitoring focused on clients’ sense of wellbeing. Symptom management is only one piece of the puzzle in mental health treatment. For example, a client may endorse decreased symptoms of depression on the PHQ-9 (Kroenke et al., 2001), and may also report better sleep, less fatigue, and improved mood without necessarily endorsing an increased sense of wellbeing in their life (Owen et al., 2015). That client may still report a sense of lacking or languishing that the clinician may be able to attend to help bolster the client to re-engage with meaningful values, connections, and activities. As such, we encourage mental health providers to utilize this measure alongside symptom-based measures to gain a fuller picture of clients’ overall psychological functioning. In doing so, this measure can be easily administered before every session to track progress, which is consistent with best practice guidance from APA (see Boswell et al., 2023).

Our findings should be interpreted considering several limitations. First, although the single-factor model demonstrated strong CFI and SRMR values in Study 2, its RMSEA exceeded conventional benchmarks. Indeed, RMSEA can perform poorly in models with very few degrees of freedom (Kenny et al., 2015). Therefore, the collective pattern of fit indices should be considered rather than any single index. Nevertheless, replication across additional non-clinical samples is warranted. Second, internal consistency was lower in the clinical telehealth sample than in the development and non-clinical samples. Although coefficient alpha is influenced by sample characteristics and the small number of items, continued evaluation of score reliability across settings and populations is needed. Third, the RWS was moderately to strongly associated with depression and anxiety but is not intended to replace disorder-specific symptom assessment. It should instead be used alongside symptom measures when both distress and positive functioning are clinically relevant. Finally, test–retest reliability and longitudinal measurement invariance have not yet been established. Consequently, the proposed reliable-change benchmark should be regarded as preliminary pending direct longitudinal evaluation.

## Summary and conclusion

The RWS is a new ultra-brief measure of eudaimonic wellbeing designed for tracking clients’ progress throughout their course of psychotherapy. RWS consists of four items that evaluate four domains of eudaimonic wellbeing: connectedness, daily functioning, purpose, and emotion regulation. In the present study, the RWS demonstrated good internal reliability across samples using only four items to assess eudaimonic wellbeing. The RWS can be efficiently administered and scored alongside other measures of symptomatology to balance deficit-focused understanding of mental health.

## Data Availability

The datasets presented in this article are not readily available because data inquiries can be directed to Owen. There are current restrictions to accessing data due to confidentiality constraints. Requests to access the datasets should be directed to JO, jesse.owen@du.edu.

## Footnotes

1 Clinical cut-off equation = (SDclincal X SDnonclinical) + (Mclinical X Mnonclinical)/ (SDclincal X SDnonclinical).
